# The complete chloroplast genome of the ephemeral plant *Isatis minima* (brassicaceae) of northwest China

**DOI:** 10.1080/23802359.2021.1977198

**Published:** 2021-09-22

**Authors:** Feng Song, Lu Jin, Xue-Jun Ge, Ying Feng

**Affiliations:** a Key Laboratory of Plant Resources Conservation and Sustainable Utilization, South China Botanical Garden, Chinese Academy of Sciences, Guangzhou, China; b College of Life Sciences, South China Agricultural University, Guangzhou, China; c State Key Laboratory of Desert and Oasis Ecology, Xinjiang Institute of Ecology and Geography, Chinese Academy of Sciences, Urumqi, China

**Keywords:** Brassicaceae, chloroplast genome, ephemeral plants, *Isatis minima*

## Abstract

The complete chloroplast genome of *Isatis minima*, a typical ephemeral plant of Brassicaceae in the Central Asia desert, was sequenced and characterized in this study. The genome 153,642 bp in size, contains a typical quadripartite genome organization including LSC and SSC regions of 83,423 bp and 17,709 bp, and two copies of the IR regions of 26,255 bp. It has 113 unique genes, including 79 protein-coding, 30 tRNA, and four rRNA genes. Phylogenetic analysis fully resolved *I. minima* in a monophyletic clade with *I. tinctoria*. This bioinformatic data contributes to the phylogenetics systematics and evolutionary history of Brassicaceae.

*Isatis* Tourn. ex L. (Brassicaceae) contains about 85 species native to the Mediterranean region east to central Asia (Mabberley 2017). There are four species in China. The roots and leaves of *Isatis* are used for medicinal purposes and are also sources of dye (Zhou et al. 2001). *Isatis minima* Bunge is a typical ephemeral plant that mainly grows in deserts, steppe, and roadsides areas of Xinjiang and Gansu (Zhou et al. 2001). In this study, we obtained the complete chloroplast (cp) genome of *I. minima* using genome skimming methods. We characterized the cp genome to determine its genome structure and provide resources for studying the phylogenetics and speciation of ephemeral plant classified in the Brassicaceae.

The specimen of *I*. *minima* was collected from Urumqi, Xinjiang Uygur Autonomous Region (43°48’57.45"N, 87°33’51.34"E), and dried with silica gel. A voucher was deposited at the Herbarium of South China Botanical Garden (IBSC, http://herbarium.scbg.cas.cn/, Xue-Jun Ge, xjge@scbg.ac.cn) under the voucher number IBSC 0812950. Total genomic DNA isolation protocols followed the cetyltrimethyl ammonium bromide (CTAB) method (Doyle and Doyle 1987). DNA extracts were fragmented for 300 bp short-insert library construction and sequenced −2 × 150 bp paired-end (PE) reads on an Illumina HiSeq X-Ten instrument at the Beijing Genomics Institute (BGI, Shenzhen, China). The complete cp genome was assembled using GetOrganelle (Jin et al. 2018), and the finished cp genome was annotated with GeSeq (Tillich et al. 2017) and adjusted manually using Geneious v 11.0.2 (Ripma et al. 2014), with *I*. *tinctoria* (MK637736) as reference. The annotated cp genome was submitted to GenBank (Accession number: MZ488447). MISA-web v2.1 (Beier et al. 2017) was applied to identify simple sequence repeats sequences (SSR).

The complete cp genome of *I*. *minima* is 153,642 bp in length (36.5% GC contents), with a large single copy region (LSC) of 83,423 bp (34.3% GC contents), a small single copy region (SSC) of 17,709 bp (29.5% GC contents), and a pair of two inverted repeats regions (IR) of 26,255 bp (42.7% GC contents). The cp genome encoded 113 unique genes, including 79 protein-coding, 30 tRNA, four rRNA genes. The cp genome generated in this study exhibited typical plastome structure, gene order and GC content was similar to that of *I. tinctoria* (Yang and Wang 2017). A total of 68 SSRs were detected, 59 of which were mono-nucleotide (A/C/T, 86.76%), eight were di-nucleotides (AT/TA, 11.76%), one was tetra-nucleotide repeat (TTAA, 1.47%), respectively.

A phylogenetic analysis of the Brassicaceae was carried out on 31 complete cp genomes, and *Tarenaya hassleriana* (*Tarenaya*, Cleomaceae, Accession number: NC_034364.1) was designated as the outgroup. The DNA sequences for these 32 complete cp genomes (after removing one IR) were aligned using the default option implemented in MAFFT version 7 (Katoh and Standley 2013). The GTR + G model was used for Maximum likelihood (ML) tree analyses using RAxML version 8.2.10 (Stamatakis 2014) and then was generated with 1000 replicates. Consistent with Li et al. (2014), the species of *Isatis* formed a monophyletic clade with 100% bootstrap support value (Figure 1) and shared a sister relationship to *Conringia planisiliqua* (Accession number: NC_049619).

**Figure 1.**
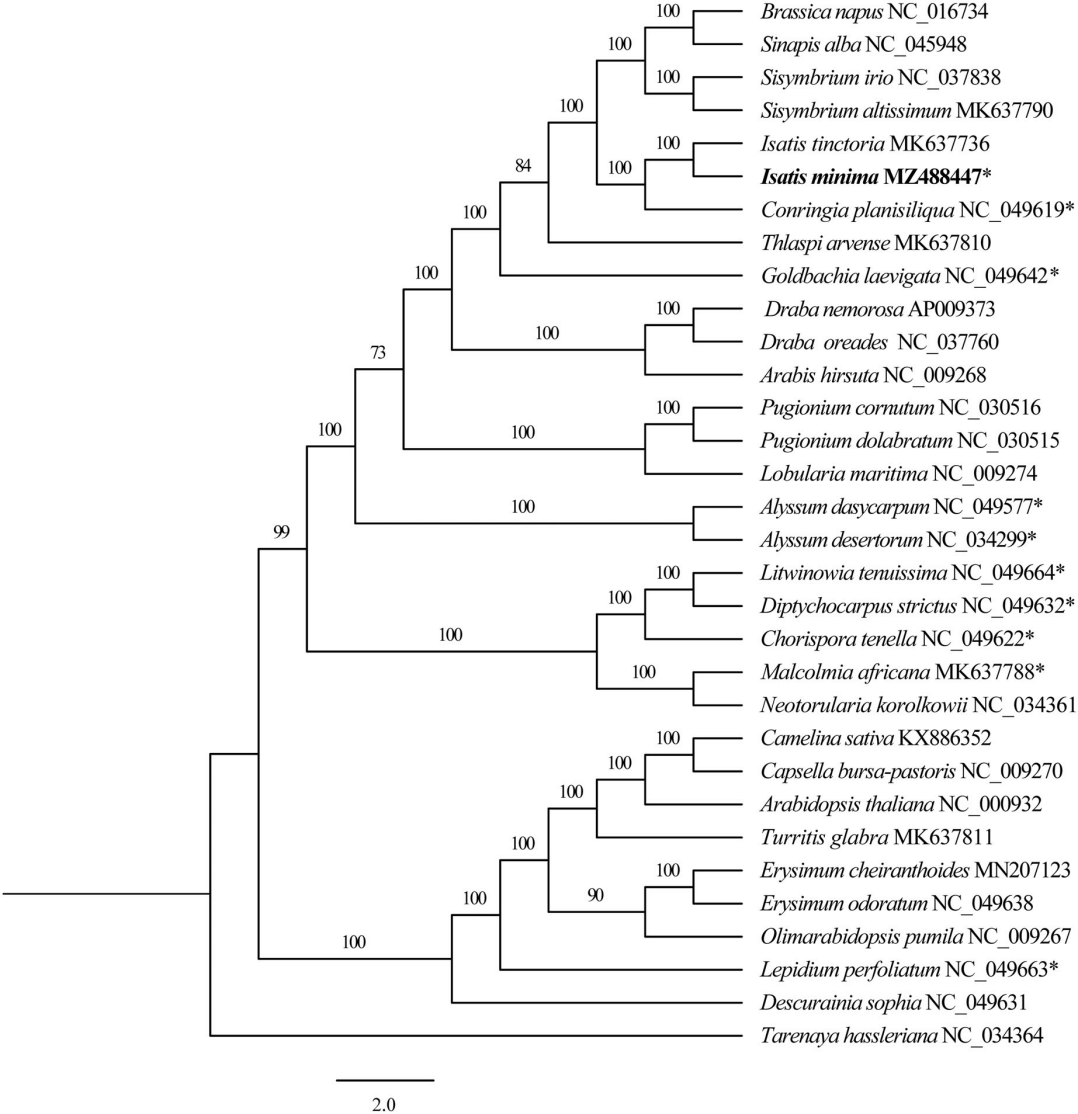
The maximum-likelihood (ML) phylogenetic tree based on 31 complete chloroplast genomes (only one IR region) of Brassicaceae (*Tarenaya hassleriana* was chosen as outgroup). ‘*’ indicates ephemeral plants. The number above branches indicated the value of bootstrap values.

## Data Availability

The genome sequence data that support the findings of this study are openly available in GenBank of NCBI at (https://www.ncbi.nlm.nih.gov/) under the accession no. MZ488447. The associated BioProject, SRA, and Bio-Sample numbers are PRJNA742888, SRS9358470, and SAMN19986842 respectively.
